# Global transcriptome analysis of spore formation in *Myxococcus xanthus *reveals a locus necessary for cell differentiation

**DOI:** 10.1186/1471-2164-11-264

**Published:** 2010-04-26

**Authors:** Frank-Dietrich Müller, Anke Treuner-Lange, Johann Heider, Stuart M Huntley, Penelope I Higgs

**Affiliations:** 1 Department of Ecophysiology, Max Planck Institute for Terrestrial Microbiology, 35043, Marburg, Germany; 2 Institute for Microbiology and Molecular Biology, University of Giessen, 35392 Giessen, Germany; 3 Laboratory for Microbiology, Department of Biology, Philipps University Marburg, 35043, Marburg, Germany; 4 Current address: Department of Microbiology, Ludwig Maximilians University Munich, 82152, Planegg-Martinsried, Germany; 5 Current address: Department of Ecophysiology, Max Planck Institute for Terrestrial Microbiology, 35043, Marburg, Germany

## Abstract

**Background:**

*Myxococcus xanthus *is a Gram negative bacterium that can differentiate into metabolically quiescent, environmentally resistant spores. Little is known about the mechanisms involved in differentiation in part because sporulation is normally initiated at the culmination of a complex starvation-induced developmental program and only inside multicellular fruiting bodies. To obtain a broad overview of the sporulation process and to identify novel genes necessary for differentiation, we instead performed global transcriptome analysis of an artificial chemically-induced sporulation process in which addition of glycerol to vegetatively growing liquid cultures of *M. xanthus *leads to rapid and synchronized differentiation of nearly all cells into myxospore-like entities.

**Results:**

Our analyses identified 1 486 genes whose expression was significantly regulated at least two-fold within four hours of chemical-induced differentiation. Most of the previously identified sporulation marker genes were significantly upregulated. In contrast, most genes that are required to build starvation-induced multicellular fruiting bodies, but which are not required for sporulation *per se*, were not significantly regulated in our analysis. Analysis of functional gene categories significantly over-represented in the regulated genes, suggested large rearrangements in core metabolic pathways, and in genes involved in protein synthesis and fate. We used the microarray data to identify a novel operon of eight genes that, when mutated, rendered cells unable to produce viable chemical- or starvation-induced spores. Importantly, these mutants displayed no defects in building fruiting bodies, suggesting these genes are necessary for the core sporulation process. Furthermore, during the starvation-induced developmental program, these genes were expressed in fruiting bodies but not in peripheral rods, a subpopulation of developing cells which do not sporulate.

**Conclusions:**

These results suggest that microarray analysis of chemical-induced spore formation is an excellent system to specifically identify genes necessary for the core sporulation process of a Gram negative model organism for differentiation.

## Background

Many bacteria have evolved the ability to survive long-term environmentally unfavourable conditions by differentiation into dormant resting stages, most commonly referred to as spores or cysts. Production of resting stages involves major rearrangements of many fundamental growth and cell-cycle processes and thus represents an important tool for understanding core physiological mechanisms themselves. Resting stage differentiation in prokaryotes is phylogenetically diverse and, not surprisingly, there is a large variety in the mechanisms employed [1].

The mechanism of sporulation has been most intensively investigated in Gram positive species such as *Bacillus subtilis *which forms endospores in response to unfavourable environmental conditions. These spores arise from an asymmetric cell division event which produces a prespore that becomes engulfed by, matured within, and then released from, the mother cell [2]. The complex, multi-layered spore surface which is important for much of the endospore's extraordinary resistance properties is assembled onto the pre-spore surface within the protective compartment of the mother cell [3].

Most of the Myxobacteria, a group of Gram negative soil inhabiting delta proteobacteria, are known to produce spores in response to starvation. The spores of *Myxococcus xanthus*, the best characterized of the myxobacterial species, are produced by remodelling the rod-shaped cell into a sphere in the absence of a cell division event. The resulting spores are resistant to up to 60°C heat, desiccation, UV-irradiation, sonication, detergents and enzymatic digestion [4]. Thus, the *M. xanthus *sporulation mechanism is significantly different from that of endospore formation and serves as an important model for alternate prokaryotic differentiation processes.

Because *M. xanthus *is a predatory bacterium whose growth is facilitated by high population density, the cells have evolved to sporulate in mounds to aid in dispersal of groups of cells [5,6]. Differentiation into spores is thus coupled to an approximately 72 hour starvation-induced multicellular developmental program [7] in which cells are first directed to aggregate into mounds of approximately 10^5 ^cells, and spore differentiation is induced at the completion of aggregation and only within these mature mounds (fruiting bodies). The developmental program also includes cells that follow different cell fates including lysis (~80% of the initial population), and formation of peripheral rods which remain outside of the fruiting bodies and do not sporulate [8-10]. Thus, sporulation occurs in a minor proportion of the starving cells and is indirectly dependent upon multiple factors necessary to produce fruiting bodies, such as a threshold population density, motility, and a complex cascade of inter- and intra-cellular signals (reviewed in [11]). It is, therefore, difficult to identify processes that are specific for only the core sporulation mechanism. These factors impede analysis of the core sporulation process via global gene or protein expression analyses.

It is also possible, however, to artificially induce spore-like entities in exponentially growing nutrient-rich broth cultures by addition of certain chemical compounds, such as 0.5 M glycerol [12], 0.7 M dimethyl sulfoxide [13,14], beta-lactam antibiotics, and D-amino-acids [15]. In particular, addition of 0.5 M glycerol induces differentiation of virtually all of the cells in the culture into spores within 8 hours [12]. Glycerol-induced spores are not identical to starvation induced spores; mainly, they have a considerably thinner spore protective layer [16,17], and they lack certain proteins that have been previously identified in starvation induced spores (*ie*. the surface spore coat proteins S and C) [18,19], and the internal poly-phosphate storage Protein W [20]). Furthermore, in contrast to starvation induced spores in which the genome is duplicated, glycerol-induced spores have a variable genome copies likely due to the replication state of the vegetative cells at the time of glycerol addition [21]. Importantly, however, glycerol-induced spores share several key features with starvation-induced spores including morphogenesis into spherical entities that are phase bright under light microscopy, resistance to heat and ultrasound treatment, and the ability to germinate into rod-shaped vegetative cells [4]. Thus, although artificial induction of sporulation seems to have fallen out of favour as a tool [22], it clearly remains a good model system for investigation of the core mechanisms related to differentiation of vegetative rods into resistant, quiescent entities. In particular, because the glycerol-induced sporulation process is synchronized and bypasses the complex signalling pathways required for fruiting body formation, it is an excellent system in which to employ global gene profiling analyses to identify genes essential for the core differentiation process.

In this study, we performed global transcription analysis on a time course of a chemically (glycerol)-induced sporulation process with three goals: 1) to determine whether microarray analysis of the chemically-induced sporulation process can be employed as a useful tool to understand the starvation-induced sporulation process, 2) to obtain an overview of the cellular processes that are regulated during sporulation, and 3) to identify specific genes necessary for the core sporulation process. We report that 22% of the genes represented on the microarray chip were significantly regulated relative to vegetative cells. Most of the known sporulation marker genes were significantly upregulated in our data set. Analysis of the functional categories of the regulated genes suggested large proportions of the genes involved in energy metabolism, protein synthesis and fate, and in two-component signal transduction are regulated. Using the microarray data, we identified a locus of eight genes that are necessary for production of viable spores under both glycerol- and starvation-induced development, which we termed *nfsA-G*. Our data suggest these genes are transcribed together in an operon, and a P_*nfsA*_::mCherry fusion demonstrated *nfs *is expressed within fruiting bodies during starvation induced-development, but not in peripheral rods. Consistently, *nfs *expression is altered in mutants of several key developmental regulators necessary for appropriate expression of sporulation genes.

## Results and Discussion

### Microarray analysis of glycerol-induced spore formation in *Myxococcus xanthus*

To identify genes that function specifically during *M. xanthus *sporulation, we performed microarray analysis on a time course of the glycerol induced sporulation process using microarray chips generated by The Institute for Genomic Research [(TIGR), now the J. Craig Venter Institute (JCVI)] in which of 6 687 of the 7 380 *M. xanthus *protein coding genes assigned by JCVI (90.6%) are represented in triplicate analyzable spots of 70-mer single stranded DNA oligomers. Microarray analysis was performed using Cy™3/5-labelled cDNA generated from cells harvested 0.5, 1, 2, and 4 hours after addition of glycerol and was referenced to cDNA prepared from vegetatively growing cells just prior to addition of glycerol. Genes called significantly regulated were selected by a delta value from SAM analysis where the false discovery rate was 5% in combination with a two-fold cut-off criteria in at least one time point.

**Figure 1:**
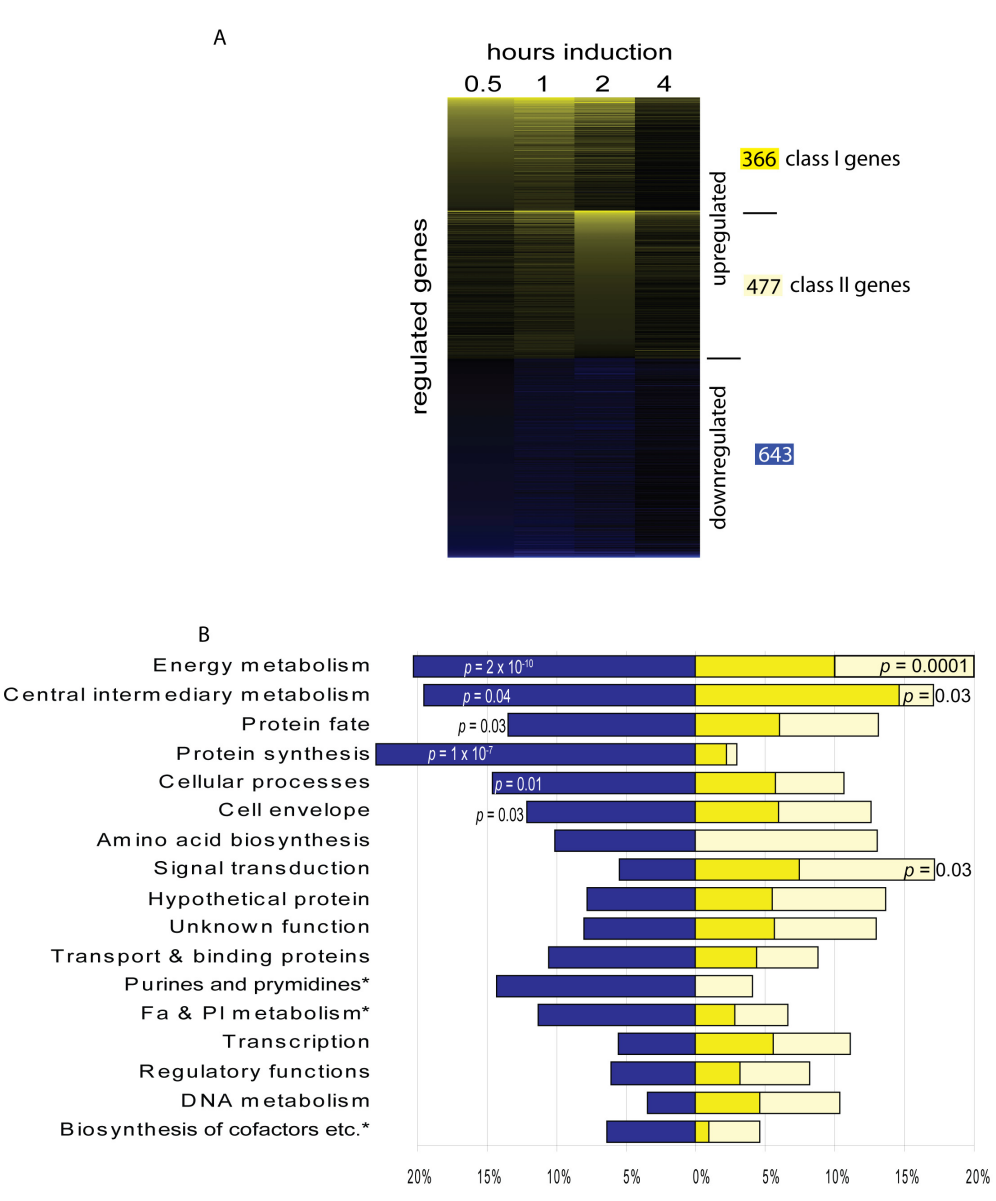
**Twenty percent of the protein coding genes are significantly regulated during glycerol-induced sporulation**. A. A heat map displaying average fold-changes of 1 485 genes whose expression is significantly regulated in at least one time point ≥ two-fold above (yellow) or below (blue) vegetative cells at the indicated hours after induction with glycerol. Upregulated genes were first organized into two self-organizing maps of genes whose expression peaks early (class I) and late (class II) and sorted by descending fold induction at 0.5 and 2 hours, respectively. Downregulated genes were sorted by descending fold induction at 0.5 hours. The number of class I and class II upregulated and downregulated genes is indicated to the right according to the colour scheme in B. B. Functional characterization of gene expression patterns. The percent of significantly down- (blue), class I up- (yellow), and class II up- (orange) genes in the listed JCVI main role categories. Role assignments were modified as described in Materials and Methods. *: main role abbreviations: Fa & Pl metabolism: Fatty acid and phospholipid metabolism; Biosynthesis of cofactors etc.: Biosynthesis of cofactors, prosthetic groups, and carriers; Purines and pyrimidines: Purines, pyrimidines, nucleosides, and nucleotides.

Analysis of the resulting microarray data revealed a total of 1 486 significantly regulated genes, corresponding to 22% of the analyzable orfs included on the chip. Of these regulated genes, 843 (13%) were upregulated and 643 (10%) downregulated. To validate the microarray data, the expression patterns of certain up- (*sigB, sigC, mspC, prU*), down- (Mxan_5543, *atpE*) and un-regulated (*devR*) genes were confirmed by real-time PCR (Additional file 1). The 843 significantly upregulated genes were next grouped into self organizing maps in which genes were clustered based on similar transcriptional expression patterns. Our analysis revealed two patterns of expression: genes whose expression peaked either at 0.5 - 1 hour (class I; 366 genes) or at 2-4 hours (class II; 477 genes) after induction with glycerol. These results are summarized in heat maps (Figure 1A) and in Additional file 2.

### Regulation patterns of known sporulation markers

To determine whether microarray analysis of the glycerol-induced sporulation process could be a useful tool to understand the core sporulation process, we first examined whether genes encoding proteins or activities previously described to be involved in glycerol-induced sporulation displayed the predicted regulation patterns in our microarray data. Genes were designated as markers if when mutated they failed to produce glycerol-induced spores, if the respective proteins were shown to be upregulated, or if the respective enzyme activities had been demonstrated as increased during glycerol induced sporulation (Table 1). Five previously described genes yield a significant glycerol-induced sporulation defect when mutated: four different genes coding for proteins that, as members of the enhancer binding protein family, are involved in transcriptional regulation (*nla4*, *nla6, nla18*, and *nla24*) [23], and one gene (*exo*) encoding for a homolog of a polysaccharide export protein [24]. Of these genes, only *nla6 *is significantly upregulated in our data (Table 1). *exo *(Mxan_3227) was considered an unreliable spot on the microarray chip, but subsequent real-time PCR analysis confirmed that the gene was indeed upregulated (data not shown), as has been previously demonstrated using a *lacZ *fusion reporter [24]. The remaining three markers which were not significantly regulated (*nla4*, *nla18*, *nla24*) also displayed mutant vegetative growth or motility phenotypes [25-27], suggesting these genes could be constitutively expressed.

**Table 1:** Transcriptional regulation patterns of glycerol-induced sporulation markers.

marker	Mxan	Sporulation:		Detected in:		**Max. reg**.	Class	**Ref**.
					
		S	G	S	G			
**Phenotype**								

*exo*	3227	N	N	Y	Y	NA*	I*	[24]
*nla4*	2516	N	N	NT	NT	NR		[23]
*nla6*	4042	N	N	NT	NT	20.6	I	[23]
*nla18*	3692	N	N	NT	NT	NR		[23]
*nla24*	7440	N	N	NT	NT	NR		[23]

**Protein (gene)**								

S1 (*ops*)	5430	Y	Y	Y	Y	2.7	II	[28,85]
U (*pru*)	3885	Y	NT	Y	Y	93.5	II	[29]
W (*prw*)	2491	Y	NT	Y	N	NR		[20]
S2 (*tps*)	5432	Y	Y	Y	N	3.7	II	[28,85]
C (?)	?	NT	NT	Y	N			[19]

**Activity (gene)**								

	**putative Mxan**							

isocitrate lyase			4.1.3.1	NT	Y			[50]
(*aceA*)	6442					126.1	I	
malate synthase			2.3.3.9^a^	NT	Y			[50]
(*aceB*)	6441					36.3	I	
isocitrate dehydrogenase			1.1.1.41	NT	Y			[31]
(*icd*)	3537					-2.54		
trehalose synthesis			NT	Y				[30]
*(treS)*	3684					9.5	II	
*(treY)*	1533					12.9	I	
*(treZ)*	0541					NA		
*(otsAB)*	1192					5.4	I	

Glycerol-induced sporulation markers displaying a mutant sporulation phenotype (Phenotype), encoding proteins identified in glycerol induced spores (Protein), or associated with upregulated activities (Activity). Mxan; gene id; S: starvation induced spores; G: glycerol induced spores; Max. reg.: maximum regulation in microarray analysis; NT: not tested; Y: yes; N: no; Class: I (peak upregulation 0.5-1 hours) or II (peak upregulation was 2-4 hours); ?: gene unknown. * gene not analyzable on chip but subsequent RT-PCR analysis indicated peak 724-fold regulation at 0.5 hours after induction (map I criteria). Underlined markers displayed a transcriptional regulation pattern consistent with the prediction from the literature.

It has also been previously reported that the spore coat proteins Protein S1 (*ops*) [28] and Protein U (*pru*) [29] can be detected in glycerol induced spores, while Protein S2 (*tps*), and a putative phosphate storage protein, Protein W (*prw*) [20] are not (Table 1). Consistently, *ops *and *pru*, but not *prw*, were significantly upregulated. In contrast to previous data, however, we observed that *tps *was also upregulated 3.7-fold, suggesting ProteinS2 may be post-transcriptionally regulated. Protein C has been identified in the spore coat of starvation induced spores, and is absent from glycerol-induced spores; however, the corresponding gene has not yet been identified (Table 1). Finally, of the enzyme activities that have been specifically shown to be upregulated during glycerol induced sporulation, *aceA *and *aceB*, likely encoding isocitrate lyase (EC 4.1.3.1) and malate synthase (EC 2.3.3.9; previously EC 4.1.3.2), were highly upregulated (Table 1). Consistent with accumulation of trehalose in glycerol induced spores [30], genes involved in trehalose synthesis, (*treY*, *treS*, and *otsAB*) were all detected in the significantly upregulated genes (Table 1). Finally, a four-fold increase in NADP-coupled isocitrate dehydrogenase (EC 1.1.1.41) activity was reported increased during glycerol-induced sporulation [31], which is in contrast to the observed down regulation of the corresponding gene (*icd*; Mxan_3537). This inconsistency may be caused by post-translational regulation of isocitrate dehydrogenase activity, likely via phosphorylation/dephosphorylation [32]. Thus, all of the examined glycerol-induced sporulation markers either displayed the predicted regulation patterns (8/13) or deviated from the prediction with plausible explanations (5/13), suggesting that our global transcriptional profiling analysis yielded data that correlate with empirically derived data. Importantly, these correlations suggest that transcriptional regulation patterns can be used to predict functional importance during glycerol sporulation. An important caveat, however, is that in addition to genes whose protein products are subject to post-translational regulation, genes that function under both vegetative and sporulation phases of the life cycle (for instance, transcriptional regulators), may be overlooked.

### Regulation patterns of starvation-induced sporulation markers

An ultimate goal of this study was to generate a tool to understand the core *M. xanthus *sporulation process. Therefore, we next determined whether our global transcriptional profiling of glycerol-induced sporulation could also be correlated to the starvation-induced sporulation program. Genes previously demonstrated to be "core" starvation-induced sporulation genes were examined to see if they were significantly upregulated in our data. In the starvation-induced developmental program, induction of sporulation is normally coupled to completion of aggregation. Therefore, mutants in many genes that are not specifically involved in the sporulation mechanism (i.e. mutants that affect starvation- or population density-sensing, motility, or coordination of fruiting body formation) nevertheless produce a sporulation defect [33]. We specifically avoided non-core sporulation genes by choosing genes that, when mutated, significantly reduce the production of viable spores but do not appreciably prevent the formation of fruiting bodies (summarized in Table 2); genes described in Table 1 were not included here. Proteins MspA, MspB, and MspC were previously identified via comparative proteome analysis of spores and vegetative cells [34]. In our microarray data, *mspA *and *mspC *were upregulated, while *mspB *was not significantly regulated (Table 2). Genes *cbgA *and *fdgA *encoding a homolog of a *Bacillus sp*. sporulation protein, and a polysaccharide export protein, respectively, are both necessary to form fully resistant spores [35,36] and are both upregulated in the microarray data. Genes *actA *and *actB*, both predicted to encode response regulators of the two-component signal transduction family whose function thus far is proposed to be regulation of the level of CsgA (a key regulator of the aggregation and sporulation in the starvation-induced developmental program) [37], were upregulated in our microarray data. Interestingly, both mutants fail to produce glycerol-induced spores (data not shown). *mrpA*, encoding a histidine kinase proposed to function as a phosphatase to regulate sporulation through its cognate response regulator [38], is also significantly upregulated. The *dev *genes, which are exclusively expressed within fruiting bodies and are required for sporulation [39,40], and *aglU *which when mutated displays vegetative motility defects but is not necessary to build aggregation centres [41], were both not significantly regulated in our analyses. Therefore, the majority (9/12) of this list of core sporulation genes were significantly upregulated. These observations suggest that upregulation of genes during glycerol-induced sporulation can be used to predict function during starvation-induced sporulation.

**Table 2:** Transcriptional regulation patterns of starvation-induced sporulation markers.

Gene	Mxan_	Sporulation:		**Max**. **reg**.	Class	**Ref**.
					
		S	G			
*mspA*	2269	N	NT	3.6	I	[34]
*mspB*	2432	N	NT	NR		[34]
*mspC*	6969	N	NT	203.0	II	[34]
*cbgA*	5828	N	NT	5.7	I	[35]
*dev*	7440	N	Y	NR		[39]
*fdgA*	3225	N	NT	12.3	I	[36]
*aglU*	3008	N	NT	NR		[41]
*actA*	3213	N	N*	4.6	I	[37]
*actB*	3214	N	N*	23.2	II	[37]
*sigB*	3357	Y/N	Y	50.5	II	[64]
*sigC*	6209	N	N	140.5	II	[86]
*mrpA*	5123	N	NT	2.0	II	[38]

Starvation-induced sporulation markers which display a mutant sporulation phenotype without an associated defect in fruiting body formation. Mxan: gene id; S: starvation induced spores; G: glycerol induced spores; Max. reg.: maximum regulation in microarray analysis; NT: not tested; Y: yes; N: no; Class: I (peak upregulation 0.5-1 hours) or II (peak upregulation 2-4 hours). Underlined markers displayed a transcriptional regulation pattern consistent with the prediction from the literature;*: Müller and Higgs, unpublished

Out of interest, we also examined the expression profiles of 67 genes known to be involved in regulation of the developmental program (and which were analyzable on the chip) but which are not sporulation specific (i.e. are not listed in Tables 1 or 2), and 60 of these genes were not significantly upregulated under our analysis conditions (Additional file 2). Among the exceptions were *mrpC *and *fruA*, encoding two key developmental transcriptional regulators which function early during the starvation-induced developmental program [38,42,43]. Mutants in either *mrpC *or *fruA *fail to aggregate or sporulate. It has been determined that MrpC induces *fruA *expression [44], acts as an antitoxin to control programmed cell death [10], and together with FruA regulates genes expressed late in the developmental program [45,46]. Interestingly, we observed that Δ*mrpC *mutants are unable to form glycerol induced spores (data not shown), hinting that MrpC may also be necessary to induce core sporulation genes. Taken together, these data suggest that genes upregulated during glycerol induced sporulation represent good candidates for the core sporulation mechanism and hint that genes involved only in regulation of the developmental program are largely not significantly upregulated.

### Glycerol-induced sporulation involves major rearrangements in transcription of metabolic and housekeeping genes

To gain insight into the processes that could be upregulated during glycerol-induced sporulation, we examined which categories of genes were significantly regulated. As a general overview, we started with the main role categories assigned during annotation by JCVI which were further amended so that unassigned genes (largely hypothetical genes) were assigned to a category, and genes with multiple designations were assigned to one category based on BLAST analysis (R. Hedderich, unpublished) (Additional file 2). A detailed analysis of the regulation patterns of gene categories is limited by the current annotation, and some ambiguity and inconsistencies in gene main role assignments, but these analyses nevertheless highlight certain core processes in which large changes in regulation are observed. These analyses suggested that 40% (125/311) of the genes assigned to the main role category "energy metabolism" were significantly regulated with a probability that this proportion was obtained from chance of 2.5 × 10^-14 ^(*p *= 2.5 × 10^-14^) (Figure 1B and Additional file 2). Equal proportions (20%) of these regulated genes were significantly over-represented in the up- and down-regulated genes (*p *= 9.9 × 10^-5 ^and *p *= 1.9 × 10^-10^, respectively). Additionally, 37% (15/41;*p *= 0.03) of the genes assigned to the "central intermediary metabolism" category were significantly regulated with equal proportions down- and up-regulated.

**Figure 2:**
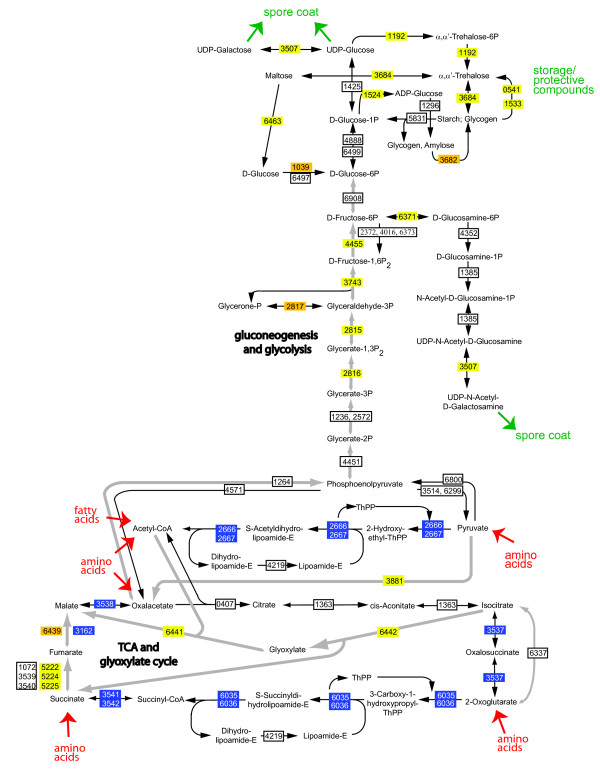
**Representation of *M. xanthus*core energy metabolism pathways significantly influenced at the transcriptional level during glycerol-induced sporulation**. *M. xanthus *metabolic pathways identified by the Kyoto Encyclopedia of Genes and Genomes (KEGG) Pathway database [47-49] were amended as described in the text and were examined for significantly regulated genes. Genes are designated as the four digit Mxan_ number. Significantly downregulated, upregulated class I, upregulated class II, and not significantly regulated genes are identified by blue, yellow, orange, and white outlined boxes, respectively. Entry sites of amino acids or fatty acids into the metabolic pathways are indicated in red. Consumption of activated sugars as precursors for spore coat components is indicated in green.

Further examination of "energy metabolism" subrole categories revealed that 35% (7/20; *p *= 1.1 × 10^-3^) and 30% (6/20; *p *= 3.0 × 10^-2^) of the genes assigned to the TCA cycle subcategory were down- and up-regulated, respectively. In addition, 39% (6/18; *p *= 1.8 × 10^-2^) of the genes assigned to the glycolysis/gluconeogenesis pathways were upregulated. Interestingly, 41% (13/39; *p *= 1.4 × 10^-4^) of the genes assigned to the "biosynthesis and degradation of polysaccharides" subrole category were upregulated. To integrate these observations, we examined which genes were significantly up- or down-regulated in the context of *M. xanthus *metabolic pathways. For these analyses, we started with the pathways assigned by the Kyoto Encyclopedia of Genes and Genomes (KEGG) Pathways database [47-49] and amended the pathways based on previous literature and data from our analyses (Figure 2). These observations strongly suggest that during glycerol-induced sporulation, *M. xanthus *cells downregulate large portions of the TCA cycle, upregulate the glyoxylate shunt, and upregulate gluconeogenesis. Most of the upregulated genes in this pathway fall into the class I category of upregulated genes (yellow bars, Figure 2) suggesting these metabolic arrangements occur early in the glycerol-induced sporulation program. These data are consistent with previous studies of specific enzyme activities [50,51]. The net effect of these metabolic actions would likely be production of precursors to carbohydrate synthesis which is important for two reasons: 1) *M. xanthus *cells do not grow on carbohydrates [31] and obtain both carbon and energy from amino acids and fatty acids [52,53], and 2) carbohydrate production increases by 200% during glycerol-induced sporulation in both soluble and insoluble fractions [51] likely corresponding to protective or storage compounds (e.g. trehalose [30] and glycogen [54]) and spore coat polysaccharides [16], respectively).

Consistent with net production of phosphoglucose units, it has been previously demonstrated that the glycerol-induced spore coat consists of 75% carbohydrate containing two-thirds N-acetyl-galactosamine and one-third glucose [16,55] and that enzyme activities involved in converting fructose biphosphate to UDP-N-acetyl-galactosamine are upregulated during sporulation [56]. The gene likely to encode the final epimerization reaction (Mxan_3507) was also significantly upregulated in our data (Figure 2). It is not known how the spore coat is added to the surface of the cell, but good candidates include a highly upregulated cluster of nine genes (Mxan_3225-3233) some of which share homology to polysaccharide export genes. Interestingly, *fdgA *and *exo *(aka Ω7536) are in this locus; *fdgA *mutants aggregate but do not sporulate [36], and *exo *has previously been characterized as a gene necessary for both glycerol and starvation-induced sporulation [24].

We also observed that three different pathways leading to trehalose synthesis were upregulated represented by *otsAB *(Mxan_1192), *treY *[Mxan_1533; *treZ *(Mxan_0541) was not analyzable on the chip], and *treS *(Mxan_3684). It has previously been demonstrated that OtsAB can convert glucose 6-P directly into trehalose [57], while TreY and TreZ convert glycogen into trehalose [58,59]. Consistent with accumulation of glycogen, a gene encoding a glucose-1 phosphate adenyltransferase (Mxan_1524) which is necessary to convert Glucose-1-P to ADP-glucose, a precursor to glycogen, is also upregulated. Finally, TreS could convert glycogen to trehalose and, furthermore, trehalose into maltose [60,61] suggesting TreS may also be necessary during spore germination.

In addition to large rearrangements of metabolic processes, analysis of over-represented functional groups in the significantly regulated genes revealed that the categories "protein synthesis- ribosomal proteins: synthesis and modification" [(25/52, *p *= 5 × 10^-21^)] and "protein fate-protein and peptide secretion and trafficking" (17/53, *p *= 3 × 10^-8^) were both over-represented in the downregulated genes (Additional file 2). In contrast, the category "protein fate-degradation of proteins, peptides, and glycopeptides" category was significantly over represented (27/136; *p *= 0.01) in the upregulated genes. Several categories representing cellular processes necessary only in vegetative growth were significantly overrepresented in the downregulated genes including "motility and chemotaxis" (13/62, *p *= 0.02), "siderophores" (5/5, p = 8 × 10^-6^), and "biosynthesis of natural products" (13/51, *p *= 0.0008) (Additional file 2). Together, these transcriptional patterns are consistent with down-shift of new protein synthesis and turn over of proteins required in vegetative growth in preparation for spore differentiation and quiescence.

We also examined the proportions of significantly regulated genes involved in regulatory processes (*i.e*. gene regulation and signal transduction). It has been suggested that in contrast to sporulation program in *B. subtilus *which is regulated primarily by cascades of sigma factor expression, the *M. xanthus *starvation-induced developmental program is regulated by cascades of signal responsive non-sigma transcriptional regulators [62]. In our analyses of glycerol induced sporulation, the "transcription-transcription factors" category was not significantly represented (11/60, *p *= 0.5) amongst the regulated genes, although the sigma 54 transcription factor (*rpoN*; Mxan_1061), *sigB *(Mxan_3357), *sigC *(Mxan_6209), as well as two extracytoplasmic function (ECF) transcription factors (Mxan_2500 and Mxan_4733) were significantly upregulated (Additional file 2). *rpoN *fell into the class I early upregulated genes, and correlates with the observation that several enhancer binding proteins (EBPs) are required for glycerol induced sporulation [23]; EBPs help RpoN form a transcription-competent open promoter complex [63]. *sigB *(and the remaining upregulated transcription factors) fell into the class II later upregulated genes, consistent with a role in spore maturation rather than spore formation [64]. Signal responsive "one component" transcriptional regulators [65], mainly lying in the "regulatory functions-DNA interactions" category, were significantly under-represented in the regulated genes (14/160; *p *= 4 × 10^-5^) (Additional file 2). However, we cannot rule out that these genes are constitutively expressed or are not highly regulated at the transcriptional level. Examination of the representation of genes involved in signal transduction demonstrated that the "signal transduction-two-component signal transduction (TCST)" category was over-represented in the upregulated genes (44/255, p = 0.03) and dispersed in both class I (19/44) and class II (25/44) upregulated genes. It is tempting to speculate that TCST systems, which contain protein members that can act as EBPs or other transcriptional regulators, play a dominant role in regulation of the core sporulation program. The relatively large family of eukaryotic-like serine/threonine kinase genes (predominant in the "regulatory functions-protein interactions" category), were not significantly represented (23/106, *p *= 0.9) in the regulated genes (Additional file 2).

The most interesting aspect of spore formation in *M. xanthus *is the reorganization of the rod-shaped cell to form a spherical spore. Control of shape transition is not understood, and none of the genes encoding proteins putatively necessary for the rod shape (eg. *mreB *and *rodA*) are significantly regulated in our analysis, although we have observed that inhibition of the function of the cell cytoskeleton protein MreB prevents glycerol induced spore formation (E. Cserti, F. Müller, and P. Higgs, unpublished observations). Intriguingly, it was recently demonstrated that peptidoglycan is degraded during glycerol-induced sporulation [66]. Examination of the microarray data indicated that genes encoding peptidoglycan synthesis enzymes [such as PBP1A (Mxan_5911), PBP1C (Mxan_2419), DacB (PBP4; Mxan_1070) were significantly upregulated (Additional file 2), and we speculate that the cells likely first rearrange peptidoglycan during shape transition from rod to sphere and that peptidoglycan may be subsequently degraded after the spore coat is in place; these hypotheses are currently being investigated in our group.

### Identification of the *nfs *cluster necessary for efficient production of viable spores

**Figure 3:**
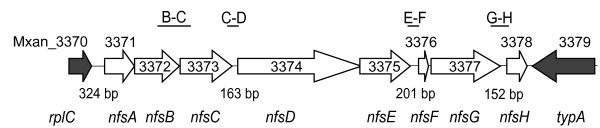
**Genetic organization of the *nfs *genes**. Schematic of the orientation and relative size of the *nfs *genes (white arrows) characterized in this study. Gray arrows depict the flanking genes. Intergenic regions are depicted by a line and the associated number of base pairs (bp) are indicated below the respective regions. Gene locus (Mxan_) ids are indicated above or within the respective genes and common names are depicted in italics below. Start codons for *nfsD *and *nfsF *were amended from the published sequence as indicated in the text. Straight lines above the genes represent regions amplified by RT-PCR during operon mapping in Figure 5).

A third goal of our studies was to identify novel genes which are specifically necessary for the core sporulation process. To this end, we searched the significantly upregulated genes for candidates using the following criteria: Genes should be 1) highly upregulated (≥ 16-fold), 2) encode proteins that are predicted to localize to the cell envelope, and 3) encode proteins whose function has not been characterized (*i.e*. hypothetical proteins). These criteria were based on the rationalization that genes highly expressed are usually important structural components and that sporulation involves major rearrangements of the cell surface in a process that is largely uncharacterized in Gram negative spore formers. Approximately 20 genes fit our criteria, including several genes that lay in a single locus of eight consecutive genes (Mxan_3371 to Mxan_3378) which were encoded in the same orientation (Figure 3). Specifically, the microarray data suggested all eight genes shared the same transcriptional pattern and were at least 10-fold upregulated within 0.5 hours of glycerol induction (Table 3). The transcriptional pattern of three of these genes (Mxans 3371, 3374, and 3378) were confirmed by real-time PCR analysis (Table 3). All eight genes were annotated as hypothetical or conserved hypothetical proteins (Table 3). Finally, at least six of the eight genes were predicted to encode proteins that reside in the cell envelope (Table 3).

**Table 3:** Characteristics of the *nfs *locus.

Mxan	gene	induction		amino acids	product	motifs	**signal seq**.	localization
							
		micro-array	RT-PCR					
3371	*nfsA*	153	4096	294	hypothetical		+	OM/C/P
3372	*nfsB*	17		442	hypothetical		+	OM
3373	*nfsC*	78		512	hypothetical		+	OM
3374	*nfsD*	55	32768	1202*	TPR repeat	TM, TPR x3	-	C/P
3375	*nfsE*	51		499	putative A-motility protein	TPR x6	+ (II)	P
3376	*nfsF*	87		94*	hypothetical		+	C/P
3377	*nfsG*	64		682	FHA/TonB domain-	FHA	-	P
3378	*nfsH*	36	4096	200	hypothetical		+	OM

Bioinformatic analysis of the *nfs *locus. Motifs were predicted by SMART [87,88]. TM, transmembrane region; TPR, tetratrico peptide repeat domain involved in mediation of protein-protein interactions [69]; FHA, Fork head-associated domain involved in binding to phospho-threonine containing proteins [70]. Localization was predicted by CELLO [74]. OM: outer membrane; C, cytoplasmic; P, periplasmic. * based on amended start codon positions (see text).

To determine if these genes were indeed necessary for glycerol induced sporulation, we generated a complete in-frame deletion of Mxans_3371-3378 in the DK1622 wild type background, generating strain PH1200. To assay for viable spore production in response to glycerol-induction, we induced both PH1200 and DK1622 cultures with glycerol for 24 hours, subjected them to heat and sonic disruption, and examined the remaining cells by phase contrast light microscopy. While the wild type culture produced phase bright spherical spores, no spores or cells could be detected in the mutant (data not shown). Consistently, when equivalent proportions of the treated cultures were incubated for 7 days on rich media plates, the mutant formed only 0.001% of the wild type colonies arising from the germinating spores. We could show that this mutant phenotype arose from the deletion of the *nfsA-H *genes and was not due to a secondary effect because of the following reasons: 1) two additional independently generated mutants bearing the same in-frame deletion displayed identical phenotypes (data not shown), 2) polar effects would not be observed since the adjacent downstream gene is orientated in the opposite direction (Figure 3) and is not expressed during glycerol induced sporulation as assayed by real-time PCR (data not shown), and 3) in-frame deletions of each single gene in the locus generated the same mutant phenotype (manuscript in preparation). Thus, one or more of the genes in the locus are necessary for glycerol-induced sporulation.

**Figure 4:**
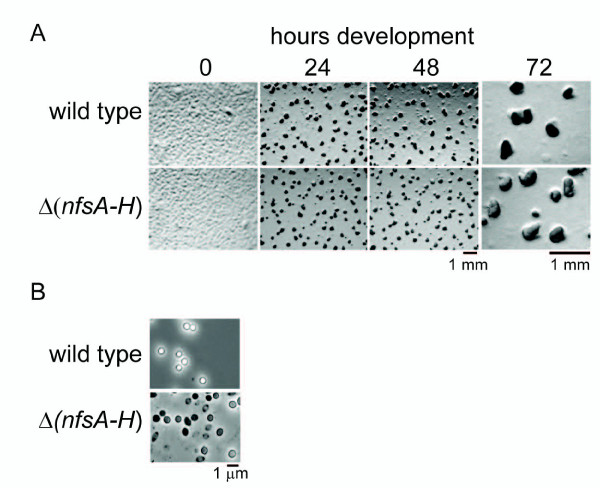
**The *nfs *locus is necessary for formation of viable starvation-induced spores**. A. Starvation induced developmental phenotype of the wild type (DK1622) and Δ(*nfsA*-*H*) (PH1200) strains. 4 × 10^7 ^cells were spotted onto nutrient limited CF agar plates, incubated at 32°C, and development was recorded at the indicated times. By 24 hours development cells aggregate into mounds (fruiting bodies) of approximately 10^5 ^cells. Fruiting bodies are displayed at higher magnification at 72 hours to illustrate that the Δ(*nfsA*-*H*) fruiting bodies fail to darken. B. Heat and sonication resistant spores (wild type; DK1622) and entities [Δ(*nfsA*-*H*); PH1200] isolated after 120 hours of development from A and examined by phase contrast microscopy.

To determine whether the deletion mutant is also perturbed in starvation induced sporulation, the mutant was induced to develop on nutrient-limited CF agar plates. The mutant began to aggregate exactly as wild type and formed similar looking aggregates from 0-24 hours (Figure 4A). However, by 48 hours, the wild type fruiting bodies began to darken with the onset of sporulation, whereas the mutant fruiting bodies failed to darken even after continued incubation for 120 hours (Figure 4 and data not shown). After 120 hours, harvested cells were subject to heat and sonic disruption, and enumerated using a hemacytometer. While the wild type produced 2.2 ± 0.3 × 10^7 ^heat and sonication resistant entities, the mutant produced 6.3 ± 1 × 10^6 ^entities (28 ± 16% of wild type) which, when examined by phase contrast light microscopy, were non-refractile, slightly misshapen spheres compared to the spherical refractile wild type spores (Figure 4B). Germination ability was assayed by plating both wild type and mutant on nutrient rich agar, the mutant produced 5 ± 34% of wild type germinating spores.

When assayed for development under strict starvation submerged culture conditions, the mutant behaved similarly as on nutrient limited CF plates: mutants cells aggregated with the same timing as wild type, but the fruiting bodies failed to darken (data not shown). Interestingly, under submerged culture conditions, the mutant formed 103 ± 12% of wild type heat and sonication resistant spores (2.9 ± 0.7 × 10^6 ^and 2.8 ± 0.4 × 10^6 ^spores, respectively). However, the non-refractile misshapen mutant entities only germinated at 3 ± 16% of wild type levels (wt: 3.5 ± 0.6 × 10^6^; Δ*nfs*: 9.3 ± 2 × 10^4 ^germinating spores) (data not shown). Together, these results indicate that one or more of the eight genes in this newly identified locus are specifically necessary for the core sporulation process. We therefore propose that the eight genes in this locus should be designated *nfs *(necessary for sporulation) *A-H*.

### *nfsA-H *consist of a single transcriptional unit

The observations that the eight *nfs *genes are oriented in the same direction and our finding that they share the same glycerol-induced expression pattern suggested that these genes could comprise a single transcriptional unit. Careful examination of the gene sequences in this locus strongly suggested that currently annotated start codons for *nfsD *and *nfsF *were likely incorrect. In *nfsD*, the GTG start codon is not preceded by a sequence that could fit to a Shine-Dalgarno ribosome binding site, whereas an ATG codon 48 bp downstream [base pair (bp) 3918630 of the published genome sequence] is preceded by an appropriately positioned GCCAGGAGC sequence. In *nfsF*, the start codon is predicted to be TTG, unlikely in the GC rich *M. xanthus*, whereas the third codon downstream is an ATG (bp 3923942).

**Figure 5:**
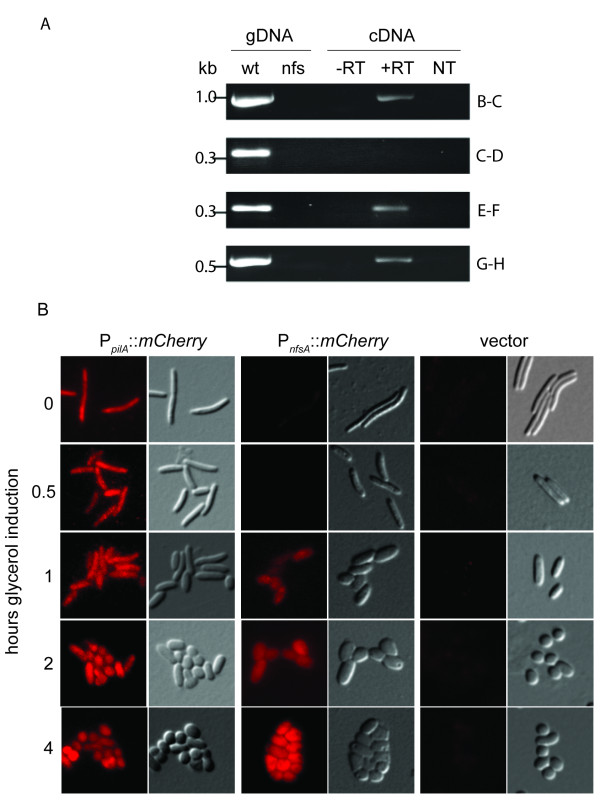
**A promoter element upstream of *nfsA *likely drives expression of *nfsA-H***. A. Reverse transcriptase PCR analysis on the intergenic regions (indicated by lines in Figure 3) between *nfsB *and *nsfC *(B-C), *nfsC *and *nfsD *(C-D), *nfsE *and *nfsF*, (E-F), and *nfsG *and *nfsH *(G-H). PCR products were amplified from genomic DNA (gDNA) from wild type strain DK1622 (wt) or Δ(*nfsA*-*H*) strain PH1200 (*nfs*), and from cDNA generated in the absence (-RT) or presence (+RT) of reverse transcriptase, or from a mock cDNA reaction in which RNA was omitted (water). RNA from cells induced with glycerol for 0.5 hours was used to generate cDNA. B. Expression of mCherry reporter constructs during glycerol induced sporulation. Strains expressing mCherry fused after the promoter from *pilA *(P_*pilA*_::*mCherry*; strain PH1221), the 500 bp upstream of *nfsA *(P_*nfsA*_::*mCherry*; strain PH1220) or the vector backbone (vector; strain PH1222) were induced with glycerol, harvested at the indicated time points, and examined by fluorescence (left images) or DIC (right images) microscopy.

Based on differential gene transcription patterns between the upstream Mxan_3370 gene and *nfsA*, a promoter likely exists upstream of *nfsA*. However, significant intergenic regions could be identified between *nfsB *and *C *(23 bp), *nfsC *and *D *(214 bp), *nfsE *and *F *(207 bp), *nfsF *and *G *(55 bp), and *nfsG *and *H *(152 bp). As per the current annotation, we could not identify convincing open reading frames in these intergenic regions. To determine if these genes are co-transcribed, we attempted to PCR amplify these intergenic regions using cDNA generated from 30 min glycerol-induced cells. PCR products could be detected from all of these intergenic regions except between *nfsC *and *nfsD *(Figure 5A). Control reactions in which wild type genomic DNA was used as a template produced the expected PCR product in all reactions; no PCR products could be detected using Δ*nfsA-G *genomic DNA or when mock cDNA samples in which either the reverse transcriptase or template RNA was omitted was used as template. These results suggest two putative promoters in the *nfs *locus: upstream of *nfsA *and upstream of *nfsD*.

To further examine whether these two putative promoters could drive gene expression, we generated P_*nfsA*_::*mCherry *and P_*nfsD*_::*mCherry *fusions in which the 323 bp upstream of the first codon of *nfsA *(P_*nfsA*_) and 964 bp upstream of *nfsD *(P_*nfsD*_), respectively, were fused to the second codon of the gene encoding the red fluorescent protein, mCherry. These constructs were inserted into the heterologous Mx8 phage attachment (*attB*) site in the wildtype DK1622 genome, resulting in strains PH1220 and PH1227, respectively. As positive and negative controls, we also constructed strains bearing the essentially constitutively active *pilA *promoter (P_*pilA*_) fused to *mCherry *(P_*pilA*_::*mCherry*) (PH1221) and the vector backbone (without the *mCherry *gene) (PH1222), respectively. All strains exhibited the wild type glycerol-induced sporulation and starvation-induced development, indicating the respective plasmid insertions did not generate a mutant phenotype (data not shown). These strains were examined by fluorescence microscopy for mCherry production in vegetative growth conditions and at several time points after induction with glycerol. In the P_*pilA*_::*mCherry *strain, fluorescence was detected in vegetative rods and throughout the differentiation process, while in the vector control strain only low level of background autofluorescence was detected (Figure 5B). Examination of the P_*nfsA*_::*mCherry *strain revealed that fluorescence was not detected in vegetative rods, but began to be detected between 0.5 to 1 hours after induction with glycerol (Figure 5B). In contrast, no significant expression could be detected at any of the examined time points in the P_*nfsD*_::*mCherry *strain (data not shown).

Similar results were observed when the cells were induced to develop in response to starvation: specific fluorescence could be detected in the sporulating P_*nfsA*_::*mCherry *strain (described below), while only non-specific fluorescence could be detected in the P_*nfsD*_::*mCherry *strain (data not shown). These results suggest that a second promoter does not lie in the intergenic region between *nfsB *and *nfsD *and that the reverse transcriptase-PCR did not amplify this region perhaps because of interfering secondary structure in the RNA or cDNA. Thus, the *nfsABCDEFG *genes are most likely a single transcriptional unit expressed from a promoter lying within the 323 bp upstream from *nfsA*.

### *nfs *is expressed late during starvation-induced development specifically in sporulating cells

**Figure 6:**
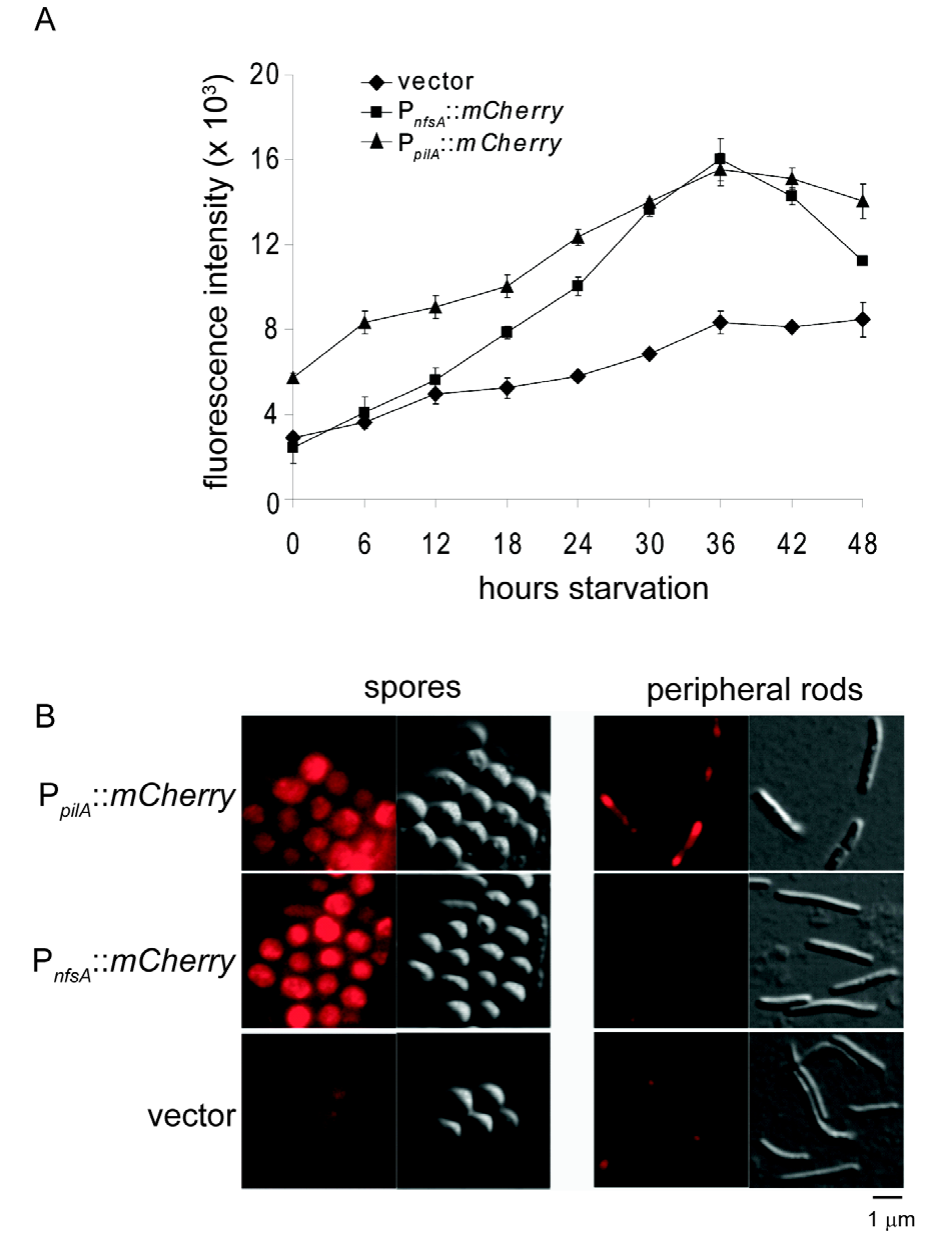
***nfs *genes are upregulated late during starvation induced development and expressed only within sporulating cells**. A. The starvation-induced developmental program was induced in submerged culture for strains expressing mCherry fused after the promoter from *pilA *(P_*pilA*_::*mCherry*; strain PH1221), the 500 bp upstream of *nfsA *(P_*nfsA*_::*mCherry*; strain PH1220) or the vector backbone (vector; strain PH1222) in the DK1622 background. At the indicated times, mCherry fluorescence was recorded in a plate reader. B. Constructs expressing mCherry fused after the promoter from *pilA *(P_*pilA*_::*mCherry*; strain PH1221), the 500 bp upstream of *nfsA *(P_*nfsA*_::*mCherry*; strain PH1220) or the vector backbone (vector; strain PH1222) in the DK1622 background were induced to develop under submerged culture for 36 hours. Peripheral rods and fruiting body spores were separated and examined by fluorescence (left images) or DIC (right images) microscopy.

To examine the expression pattern of the *nfs *mutants during starvation induced development, we next measured the relative mCherry fluorescence of the P_*nfsA*_::*mCherry*, P_*pilA*_::*mCherry *and vector control strains by developing the cells in submerged culture in dishes that could be inserted directly into a plate reader and thus measured fluorescence at various times during the developmental program. The P_*pilA*_::*mCherry *fusion was detected from 0 hours and gradually increased 2.5-fold at 36 hours of development (Figure 6A). In contrast, the P_*nfsA*_::*mCherry *fusion began to be detected between 12-18 hours of development increasing to 6.5 times at 36 hours. There was a gradual accumulation of autofluorescence in the vector control strain (Figure 6A) which may correspond to build up of exopolysaccharides.

Two cell types have been observed during the starvation-induced development program in *M. xanthus*, cells that aggregate into fruiting bodies and then differentiate into spores, and cells that do not aggregate and remain as undifferentiated peripheral rods. To examine whether both cell types expressed the *nfs *locus, the P_*nfsA*_::*mCherry*, P_*pilA*_::*mCherry *and vector control strains were developed for 36 hours, fruiting bodies were separated from peripheral rods by low speed centrifugation, and the cells from both fractions were examined by fluorescence microscopy. Both peripheral rods and spores fluoresced in the P_*pilA*_::*mCherry *strain, while in the P_*nfsA*_::*mCherry *strain, mCherry specific fluorescence could only be detected in spores (Figure 6B). Together, these results demonstrate that during the starvation induced developmental program, *nfs *is expressed late in the developmental program and specifically within the sporulating cells.

**Figure 7:**
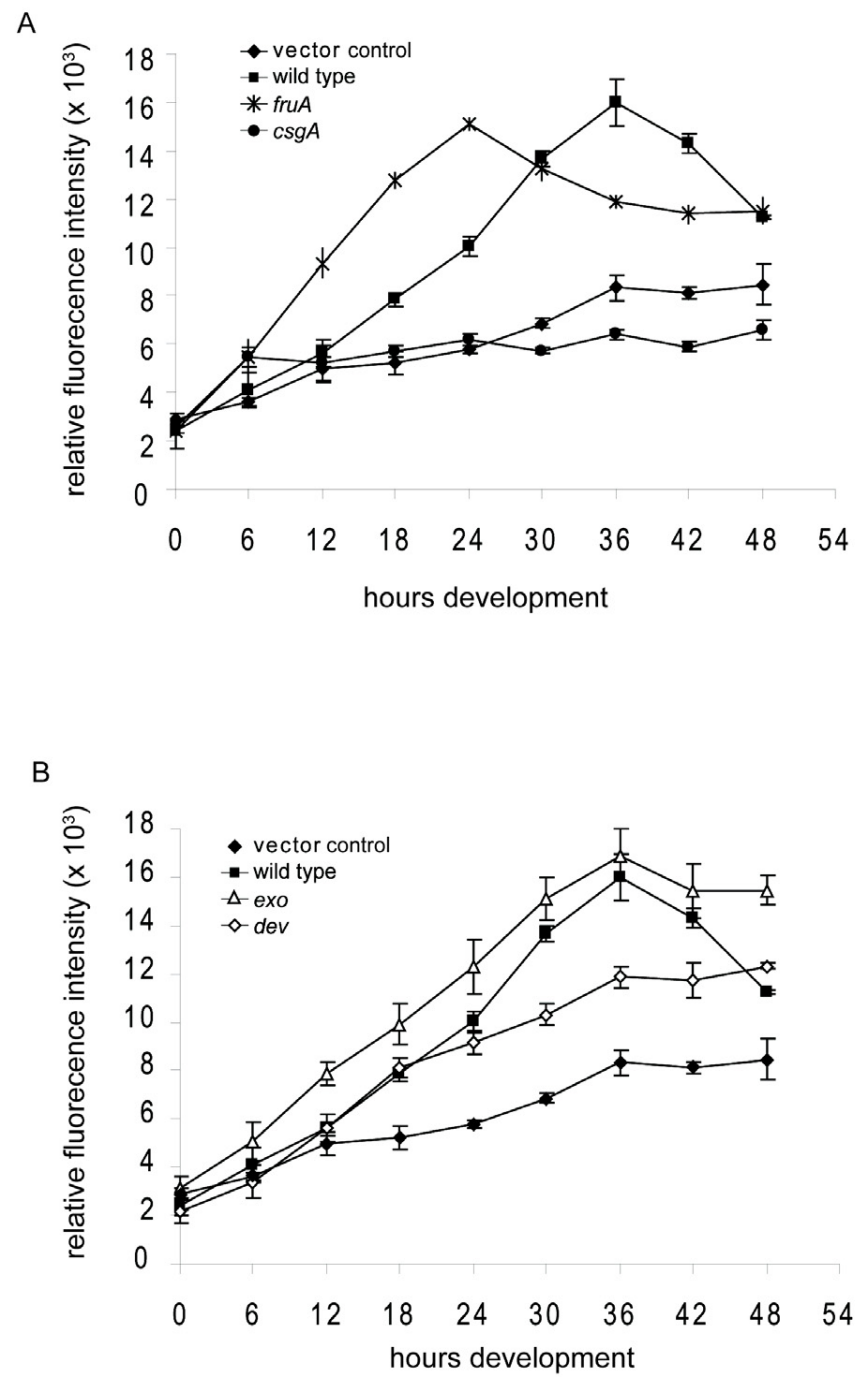
***nfs *expression is dependent on key regulators of the developmental program**. Fluorescence detected over the course of development from strains expressing the vector control (black diamonds) or *nfs *promoted *mCherry *(P_*nfsA*_::*mCherry*) constructs in the wildtype (black squares) background strains PH1222 and PH1220, respectively (A and B). A. Strains expressing P_*nfsA*_::*mCherry *in the *fruA *(stars), and *csgA *(black circles) background strains PH1224 and PH1226, respectively. B. Strains expressing P_*nfsA*_::*mCherry *in the *exo *(white triangles) and *dev *(white diamonds) background from strain PH1225 and PH1223, respectively. Strains were induced to develop under submerged culture and fluorescence was recorded by a plate reader at the indicated times.

During the starvation induced developmental program, the expression of many sporulation specific genes is dependent, directly or indirectly, on several key developmental regulators which are necessary for inducing fruiting body formation and coordinating onset of sporulation with completion of aggregation [11]. To determine if the *nfs *locus fits this class of sporulation specific genes, we next analyzed the P_*nfsA*_::*mCherry *expression pattern during starvation-induced development in strains bearing disruptions in key developmental regulators (*fruA*, *csgA*, *devR*, and *exo *(Mxan_3227). Both *fruA *and *csgA *mutants fail to form fruiting bodies and cannot sporulate [42,67]. P_*nfsA*_::*mCherry *was not expressed in the *csgA *mutant, but surprisingly, was induced earlier in the *fruA *mutant compared to the wild type background (Figure 7A). FruA is a transcriptional regulator, and these results suggest that FruA might, directly or indirectly, initially repress *nfs *expression. *dev *mutants aggregate but fail to sporulate and the *dev *genes are expressed exclusively in fruiting bodies [39,40]. In the *dev *mutant background, P_*nfsA*_::*mCherry *expression was similar to that in the wild type background from 0-18 hours, but subsequently failed to accumulate to wild type levels (Figure 7B). Finally, like *dev *mutants, *exo *mutants aggregate but cannot sporulate, and *exo *is not expressed in *dev *mutants [24]. In the *exo *background, P_*nfsA*_::*mCherry *was expressed at slightly higher levels than in wild type but followed a similar induction pattern (Figure 7B). These results are consistent with the class of genes that are expressed exclusively in fruiting bodies and dependent on the coordination of sporulation to aggregation.

In summary, our experimental data indicates that the *nfs *locus is necessary for the core sporulation process based on the following observations: 1) the Δ(*nfsA-H*) mutant is compromised in the ability to produce both glycerol- and starvation-induced viable spores, but does not affect formation of starvation-induced fruiting bodies, and has no obvious vegetative defect, 2) the locus is upregulated during both sporulation pathways, and is expressed specifically in the sporulating cell subpopulation during starvation-induced development, and 3) *nfs *expression during starvation-induced development shares a similar pattern of dependency on key starvation-induced developmental regulators as for other previously described sporulation markers [24].

These genes encode proteins that contain no previously characterized catalytic domains, and it is thus unclear what exact role the proteins play in spore formation. Although NfsE (Mxan_3225) has been annotated as "putative adventurous motility protein T", presumably because it shares some sequence similarity with a gene previously shown to be defective in adventurous motility *aglT *[68], it is not the closest paralog to *aglT*, and the Δ*nfsA-H *deletion displays no defect in adventurous (single cell) motility (data not shown). Bioinformatic analyses suggest that NfsD and NfsE contain multiple tetra-tricopeptide repeat (TPR) domains which play a role in mediating protein-protein interactions [69], and NfsG contains a forkhead-associated (FHA) domain which also mediates protein interactions to phospho-threonine containing proteins [70]. NfsA, B, C, and H contain putative signal sequences consistent with export from the cytoplasm, NfsE is predicted to be anchored in the outer membrane via a lipid attachment, and NfsD is predicted to encode an amino-terminal transmembrane segment suggesting it is anchored in the inner membrane. Furthermore, secondary structure analysis of NfsA and NfsH predicts these proteins are beta-barrels consistent with localization in the outer membrane. BLASTp analysis indicates that NfsA-H contain orthologs in the Myxobacteria species *Stigmatella aurantiaca *(Stiau_2471 to _2480) and the synteny of the locus is conserved. All together, these observations, coupled with our experimental data, suggest the Nfs proteins are located in the cell envelope and likely form a functional complex which is necessary to produce viable spores. We are currently confirming the localization and further analyzing the function of the individual proteins during both glycerol- and starvation-induced sporulation.

## Conclusions

Global transcriptional analysis of artificially-induced spore differentiation in *Myxococcus xanthus*, a Gram negative bacterium, was shown to be an excellent model system for the core sporulation program which normally occurs at the culmination of a starvation-induced multicellular developmental program. Examination of significantly regulated gene transcriptional patterns provided an overview of sporulation associated rearrangements of metabolic and physiologic processes. From the significantly upregulated gene pool, a locus of hypothetical genes was identified and demonstrated to be essential for the core sporulation process in both the artificial- and starvation-induced developmental program.

## Methods

### Strains and growth conditions

Strains used in this study are listed in Additional file 3. *M. xanthus *strains were grown vegetatively at 32°C on CTT agar plates (1% Casitone, 10 mM Tris-HCl pH 8.0, 1 mM potassium phosphate pH 7.6, 8 mM MgSO_4_, 1.5% agar) or in CTT broth (CTT lacking agar) [71]. Plates were supplemented with 100 μg ml^-1 ^kanamycin or 10 μg ml^-1 ^oxytetracycline, where necessary. *Escherichia coli *cells were grown under standard laboratory conditions in Luria-Bertani broth supplemented with 50 μg ml^-1 ^kanamycin, where necessary.

### Microarray sample preparation, probe generation and hybridization

To analyze global gene expression patterns during the *M. xanthus *glycerol-induced sporulation process, microarray analysis was performed on three independent biological replicates in which samples from 0.5, 1, 2, and 4 hours after glycerol-induction were compared to reference vegetative samples. To generate samples, *M. xanthus *DK1622 cells were grown in CTT broth at 32°C with shaking at 240 rpm on horizontal shakers. At an optical density at 550 nm (OD_550_) of 0.5, half of the culture (for reference) was removed and immediately added in 45 ml aliquots to 5 ml stop solution (5% ethanol saturated phenol pH > 7), harvested by centrifugation at 4°C, snap frozen in liquid nitrogen and stored at -70°C. The remaining culture was treated with 0.5 M glycerol (final concentration) with continued incubation as above. At 0.5, 1, 2 and 4 hours after addition of glycerol, samples were harvested as above.

Total RNA from cells harvested at each time point was isolated using the hot phenol method as previously described [72,73]. RNA was then treated with DNase I (Ambion) and purified via RNeasy column (Qiagen) according to manufacture's instructions. RNA was confirmed to be free of contaminating DNA by PCR analysis.

Microarray probes were generated as previously described in detail [73]. Briefly, RNA was reverse transcribed into cDNA using random hexamer primers (pd(N)6, Amersham) in the presence of a deoxynucleoside triphosphate mix (25 mM each dATP, dCTP, dGTP, 10 mM dTTP, 15 mM aminoallyl-dUTP), and Stratascript reverse transcriptase (Stratagene). RNA was subsequently hydrolyzed, samples were purified, vacuum concentrated, and recovered in 0.1 M sodium bicarbonate, pH 9.3. NHS-ester dyes Cy™3 or Cy™5 (Amersham) were coupled to amino groups of the aminoallyl-dUTP incorporated cDNA. After purification, labelling efficiency was determined photometricaly and the labeled probes were either used directly for hybridization or stored at -20°C.

Microarray hybridization experiments were performed as previously described in detail [73]. Briefly, *M. xanthus *oligo arrays (JCVI) were first pre-hybridized in 5 × SSC buffer (0.75 M NaCl, 75 mM sodium citrate pH 7.0), 0.1% SDS and 1% (w/v) BSA fraction V at 42°C for 2 hours, washed in water, submerged in isopropanol and dried by low speed centrifugation. Cy™3- and Cy™5-labeled probes (representing reference and sample of one particular time point) were diluted in hybridization buffer (50% formamide, 5 × SSC buffer, 0.1% SDS, 0.6 μg ml^-1 ^salmon sperm DNA), combined in equal volumes and heated at 95°C for three minutes. Probes were applied to the prehybridized arrays, covered with a LifterSlip™ (Erie scientific), mounted into a hybridization chamber and incubated submerged in a water bath for 16 h at 42°C. Arrays were washed three times in the following buffers: Buffer 1: 2 × SSC pH 7.0, 0.1% SDS; Buffer 2: 0.1 × SSC, 0.1% SDS; Buffer 3: 0.1 × SSC. Arrays were scanned with a GenePix™4000B microarray scanner using GenePix™ Pro 6.0 image analysis software (Axon instruments) at 532 nm (Cy3™) and 632 nm (Cy5™) wavelengths simultaneously.

### Microarray data analysis

Image analysis (block and spot finding, background detection) and data acquisition were performed with GenePix™ Pro 6.0 image analysis software. Each slide was checked manually for correct positioning of the grid and detection of each spot. Normalization and data analysis were carried out with Acuity 4.0 software package (Axon instruments) applying the following criteria: Normalization: Ratio based (mean ratio of medians = 1); Substance name ≠ EMPTY; Substance name ≠ NULL; F532 Median - B532 ≥ 100 OR F635 Median - B635 ≥ 100 in at least one time point.

The biological replicates were analyzed with the Significance Analysis of Microarrays (SAM) software version 2.23 (Stanford University) to screen for significantly regulated genes. The pre-filtered datasets were first analyzed using signed area regulation analysis. A median false discovery rate (FDR) of 5% (corresponding to a delta value of 0.89) was allowed to call a gene significantly regulated. The three sets of regulated genes were then combined in Acuity generating mean values for each gene at each time point. Genes that displayed a mean log ratio < -1 or > +1 (corresponding to 2-fold up- or down-regulation) in at least one time point with data present for all time points were considered significantly regulated genes. Genes whose spots are called unreliable by JCVI were subsequently removed. The microarray data was deposited in NCBIs Gene Expression Omnibus http://www.ncbi.nlm.nih.gov/projects/geo/ under the accession number GSE17912.

Genes considered significantly upregulated and analyzable were then analyzed by the self-organizing map clustering algorithm of Acuity software. To display expression patterns of the significantly regulated genes graphically, a heat map image file correlating the log ratio of expression at each time point to colour intensity, where yellow and blue colours correspond to up- and down-regulated genes, respectively, was generated.

Analysis of regulated processes was performed using the main role and subrole categories assigned to the 7200 *M. xanthus *DK1622 protein coding sequences by J. Craig Venter Institute (JCVI) (formerly TIGR). Genes for which no role category had been assigned by JCVI were termed hypothetical and genes assigned to more than one role category were examined individually, and assigned to one category based on blast analysis (R. Hedderich) (Additional file 2). 692 Mxans were that were not represented on the arrays either because they were not included on the .gal file, were subsequently documented as unreliable by JCVI, or were prefiltered out during data analysis were removed from the subsequent analyses. The number of genes either considered not significantly regulated, or significantly up-(class I), up-(class II), or down-regulated in each main role category were represented as a percent of the total genes in that category that were represented as reliable on the chip. Genes assigned to the main role categories "disrupted reading frame" and "extrachromosomal elements" were not included in the analysis. The Pearson's Chi squared test or Fischer's Exact Test (expect categories < 5) http://www.langsrud.com/fisher.htm were used to determine the probability that the proportion of regulated genes in each category could have happened by chance. The CELLO subcellular localization predictor [74]http://cello.life.nctu.edu.tw/ was used to predict the subcellular localization of the proteins encoded by each *M. xanthus *gene.

### Real-time PCR analysis

Real-time PCR was performed in a 26 μl reaction volume using SYBR green PCR master mix (Applied Biosystems) and 0.2 μM primers specific to the target gene in a 7300 Real Time PCR System (Applied Biosystems). Template cDNA for real-time PCR analysis was generated from 1 μg DNA-free RNA (prepared as described above for micrarray analysis) and reverse transcribed into cDNA using random hexamer primers (Amersham) and Superscript III reverse transcriptase (Invitrogen) in a 20 ul reaction according to manufacture's instructions. Because an endogenous control for cDNA generation (ie constitutively expressed marker gene) is not known for *M. xanthus *cells undergoing development or differentiation, real-time PCR reactions were performed using equivalent amounts of starting RNA as described previously [72,75-77]. The optimal cDNA template dilution used in real-time PCR reactions was chosen which yielded a cycle threshold (Ct) value after 25-30 cycles and was determined for each gene target individually; equivalent dilutions were used for a time course. For each cDNA sample, a control reaction was performed on an equivalent starting volume of RNA to be certain no contaminating DNA was present, and genomic DNA was used as a positive control. Each reaction was performed in duplicate, and the average Ct value from the uninduced vegetative cells (T = 0) was subtracted from the average Ct values at each time point (T = 0.5, 1, 2, and 4 hours after induction) in order to present the expression relative to the uninduced vegetative sample (T = 0). Each profile was analyzed independently at least twice.

Real-time-PCR cycle conditions were as follows: 95°C 10 min, 95°C 15 sec, 40 cycles of 60°C 1 min and 95°C 15 sec. Melting and dissociation curves were determined at 60°C 30 sec and 95°C 15 sec. Ct values for each reaction were assigned automatically by the system software (7300 System SDS software v1.2.3).

Target specific primers were initially tested for product specificity by PCR using genomic DNA as a template. Each primer pair was then tested for PCR efficiencies close to 2 by real-time PCR analysis on a standard curve generated from four 10-fold serial dilutions of genomic DNA. Average Ct values from duplicate reactions were plotted versus the log of the target copy number. PCR efficiencies (E) for each primer pair were then determined from the slope of the associated regression curve (m) using the equation: E = 10^-(1/m). Only primer pairs with efficiency close to 2 were used. Target specific primers for real-time PCR analysis are listed in Additional file 4.

### Construction of mutants

Plasmids and mutant strains used in this study are listed in Additional file 3. Complete deletion of the *nfs *locus was generated by homologous recombination using the pBJ114 kanamycin selection/*galK *counter-selection plasmid as described previously [78,79]. To generate pFM20 [pBJ114 Δ(*nfsA*-*H*)], approximately 500 bp upstream and downstream from *nfsA *and *nfsH*, respectively, were separately amplified and fused by overlap PCR. The resulting approximately 1000 bp fragment was digested with *Kpn*I and *Hind*III and ligated into the corresponding sites of pBJ114. To generate pAL4 (P_*nfsA*_::*mCherry*), pSL8 was digested with *Hind*III and *Nde*I to remove the P_*pilA*_::*gfp *insert and generate a backbone vector consisting of the kanamycin resistance selection marker and the Mx8 *attP *sequence. A 323 bp fragment containing the putative promoter sequence upstream from the fist codon of *nfsA *was PCR amplified from genomic DNA with primers containing *Hind*III and *Nde*I restriction sites and ligated into the corresponding sites in the vector backbone. The *mCherry *gene was subsequently PCR amplified from the plasmid pXCHYN-1[80] such that 5' *Nde*I and 3' *Pst*I sites were incorporated and cloned behind the *nfs *promoter region into the respective sites of the pSL8 P_*nfsA *_plasmid. To generate pAL8, pAL4 was digested with *Hind*III and *Kpn*I to remove the P_*nfsA*_::*mCherry *insert and replace it with a P_*nfsD*_::*mCherry *insert. The P_*nfsD*_::*mCherry *insert was generated by overlap PCR in which the 964 bp upstream of the reassigned predicted start codon of *nfsD *(codon 18 of the existing annotation) was fused to the second codon of the *mCherry *gene such that 5' *Hind*III and 3' *Kpn*I restriction sites were incorporated. To generate pFM17, the kanamycin resistance in pAL4 was replaced by the tetracycline resistance cassette from pSWU30. To generate pFM16 (P_*pilA*_::*mCherry*), the *pilA *promoter was first PCR amplified from pSL8 such that a 5 '*Hind*III site and a 3' region complementary to the 5' *mCherry *sequence were incorporated. Next, the *mCherry *gene was PCR amplified from pAL4 such that a 5' region complementary to the 3' end of the *pilA *promoter and a 3' *Nde*I site were incorporated. The two fragments were fused by overlap extension PCR and ligated into a *Hind*III/*Nde*I sites of pAL4. To generate the empty control vector pFM18, pSL8 was digested with *EcoR*I and *Xba*I, treated with DNA polymerase I (Klenow) to generate blunt ends and then religated. To generate pFM44 (pCR2.1-*exo*), a 311 bp internal fragment of the Mxan_3227 gene (*exo*) was amplified by PCR and cloned into PCR2.1 TOPO^® ^vector (Invitrogen).

*M. xanthus *strain PH1244 was generated by electroporating pFM44 into DK1622 and selecting for homologous recombination into the Mxan_3227 gene by resistance to kanamycin. Strains PH1220 (P_*nfsA*_::*mCherry*), PH1227, (P_*nfsC*_::*mCherry*), PH1221 (P_*pilA*_::*mCherry*), and PH1222 (vector), were generated transforming DK1622 with pAL4, pAL8, pFM16, and pFM18, respectively, such that the plasmids integrated in the genomic Mx8 phage *attB *site by site specific recombination were selected by resistance to kanamycin. Strains PH1223 (*devR *P_*nfsA*_::*mCherry*), PH1224 (*fruA *P_*nfsA*_::*mCherry*) and PH1225 (*exo *P_*nfsA*_::*mCherry*) were generated by transforming strains DK5279, DK11063, and PH1244, respectively, with pFM17, as above with oxytetracycline selection. Strain PH1226 was generated by transforming strain DK5208 with pAL4, as above with kanamycin selection. Correct integration of all plasmids was confirmed by PCR using a primers specific to plasmid and genomic DNA for integration via homologous recombination, and by primers specific to the *attB*/*attP *region for site specific recombination [81].

### Analysis of glycerol induced-development

To determine efficiency of glycerol-induced sporulation, cells were cultivated in CTT media and induced at an OD_550 _of 0.5 with glycerol to a final concentration of 0.5 M. At the indicated time points, 10 ml cells were harvested, pelleted at 4 620 × g, resuspended in 10 ml sterile water, incubated at 50°C for 2 hours, and then sonciated three times 30 pulses, output 3, 50% duty in ice water with a Branson sonifier and microtip. Surviving spores from 10 μl of the treated samples were enumerated in a Thoma cell counting chamber (Hawksley, Lancing, UK). Mutant sporulation efficiencies are represented as percent of wild type spores. Spore viability was determined by pouring 10-fold serial dilutions of heat- and sonication treated cells suspended in CTT soft agar onto CTT agar plates. The resulting colonies arising from germination of spores were counted after 5, 10 and 14 days of incubation at 32°C. Mutant spore viability was calculated as percent of wild type germinating spores.

### Analysis of *M. xanthus *starvation induced-developmental phenotypes

Starvation-induced development was assayed on nutrient-limited clone-fruiting (CF) agar plates [82], as described previously [83]. Briefly, cells were grown to mid-log phase in CTT broth, washed, resuspended to an OD_550 _of 7 in MMC starvation medium, and 10 μl of cells was spotted onto CF plates and incubated at 32°C. Developmental phenotypes were recorded at the times indicated with a Leica MZ8 stereomicroscope and attached Leica DFC320 camera. Development was induced under submerged culture conditions as described previously [84].

### Analysis of mCherry reporter fusions

Strains were developed in submerged culture in black 24-well glass bottom plates (Greiner Bio-One). At the indicated time points, fluorescence signal intensity was measured at 615 nm wavelength in a plate reader (Tecan Infinite M200). Signals from cell-free, buffer containing wells were subtracted from signals of developing cultures to calculate absolute fluorescence intensities. Proper development was checked at each time point using a stereo microscope. All experiments were carried out in triplicate.

Separation of peripheral rods from fruiting body spores was performed as described previously [9], except development was induced under submerged culture conditions, and cells were harvested at 36 hours development.

For fluorescence microscopy, cells were spotted on Agar pads (1% (w/v) agarose in A50 starvation buffer (10 mM MOPS, pH 7.2, 1 mM CaCl_2_, 1 mM MgCl_2_, 50 mM NaCl) covered with a cover slip and examined under a Zeiss Axio Imager.M1 microscope. mCherry-specific fluorescence signals were detected at 670 nm wavelength. Images were recorded with an EM-CCD Cascade 1 K (Photometrics, Tucson) camera, and images were processed with Metamorph ver7.5.

## Authors' contributions

FDM conceived of the study, participated in its design, and carried out all the experimental studies listed. ATL and JH analyzed the energy metabolism data and generated Figure 2. SMH performed supporting bioinformatic analysis including functional categorization and predicted localization of *M. xanthus *genes, and generated the microarray heat map. PIH participated in the conception and design of the study, performed the statistical analysis, and wrote the manuscript. All authors read and approved the final manuscript.

## Supplementary Material

Additional file 1 **Confirmation of microarray results by quantitative real-time PCR**. Quantitative real-time PCR analysis (black bars) of select genes designated as significantly up- (*sigB*, *sigC*, *mspC*, *prU*) or down-regulated (Mxan_5543, *atpE*), or not significantly regulated (*devR*) in the microarray data (white bars). The data are shown as levels of each transcript at the indicated times relative to the respective level in uninduced vegetative cells. cDNA was generated from equal amounts of the RNA templates used for the microarray analysis and amplified using gene-specific primers (see Materials and Methods for details). Click here for file

Additional file 2 ***M. xanthus *orf functional category assignment**. The list of *M. xanthus *protein coding genes assigned by JCVI and the associated main role/subrole assignments used in this study. Regulation patterns assigned in this study are also included. The tally of functional categories with respect to significantly regulated genes is included. Genes associated with developmental phenotypes are listed. Click here for file

Additional file 3 **Strains and plasmids used in this study**. The list of strains and plasmids and associated genotypes used in this study Click here for file

Additional file 4 **Primers used for real-time PCR analysis**. The list of primers and associated sequences used for real-time PCR analysis (Additional File 1) and reverse transcriptase PCR analysis (Figure 5) in this study. Click here for file
