# Supplementary figures and images for: Global transcriptome analysis of spore formation in Myxococcus xanthus reveals a locus necessary for cell differentiation
Source: BMC Genomics. 2010 Apr 26;11:264. doi: 10.1186/1471-2164-11-264 (PMC2875238; doi:10.1186/1471-2164-11-264)

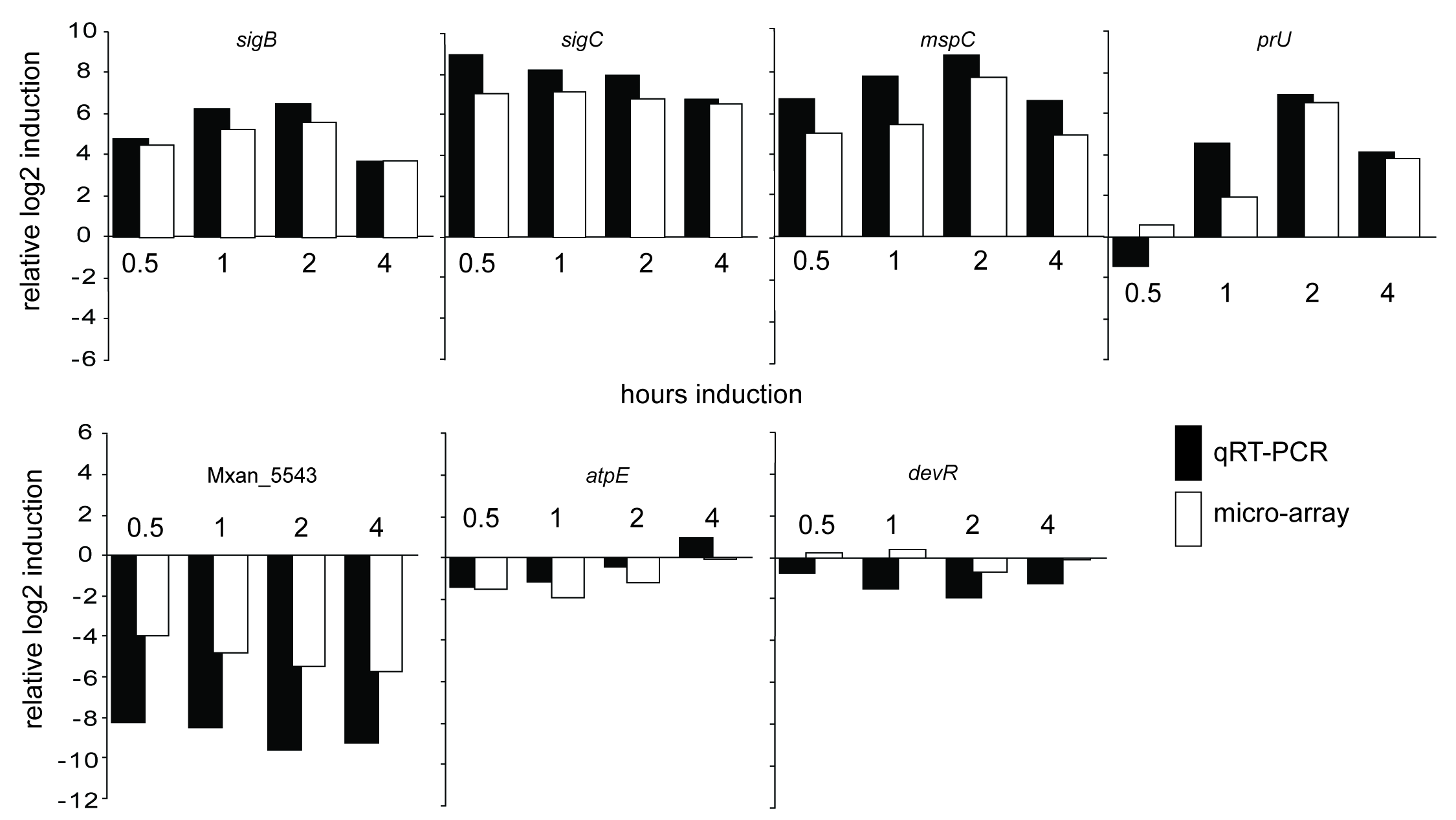

Supplement: Additional file 1 — Confirmation of microarray results by quantitative real-time PCR. Quantitative real-time PCR analysis (black bars) of select genes designated as significantly up- (sigB, sigC, mspC, prU) or down-regulated (Mxan_5543, atpE), or not significantly regulated (devR) in the microarray data (white bars). The data are shown as levels of each transcript at the indicated times relative to the respective level in uninduced vegetative cells. cDNA was generated from equal amounts of the RNA templates used for the microarray analysis and amplified using gene-specific primers (see Materials and Methods for details). [file 1471-2164-11-264-S1.TIFF]
