# Supplementary material for: Global transcriptome analysis of spore formation in Myxococcus xanthus reveals a locus necessary for cell differentiation
Source: BMC Genomics. 2010 Apr 26;11:264. doi: 10.1186/1471-2164-11-264 (PMC2875238; doi:10.1186/1471-2164-11-264)
Supplement: Additional file 3 — Strains and plasmids used in this study. The list of strains and plasmids and associated genotypes used in this study [file 1471-2164-11-264-S3.DOC]

**Additional Table 1 . Strains and plasmids used in this study**

| ***M. xanthus* strains** | **Genotype or characteristics** | **Reference or source** |
| --- | --- | --- |
| DK1622 | Wild type | [1] |
| PH1200 | DK1622 ∆(*nfsA-H*) | This study |
| PH1220 | DK1622 *attB*::pAL4 (P*_nfsA_*::*mCherry*), Km^R^ | This study |
| PH1227 | DK1622 *attB*::pAL8 (P*_nfsC_*::*mCherry*), Km^R^ | This study |
| PH1221 | DK1622 *attB::*pFM16 (P*_pilA_*::*mCherry*), Km^R^ | This study |
| PH1222 | DK1622 *attB*::pFM18 (empty vector), Km^R^ | This study |
| DK5279 | DK1622 *devR*::Ω4414, Km^R^ | [2] |
| PH1223 | DK5279 *attB*::pFM17 (P*_nfsA_*::*mCherry*), Km^R^, Tc^R^ | This study |
| DK11063 | DK1622 *fruA*::Ω7540 Tn*5lacZ*, Km^R^ | [3] |
| PH1224 | DK11063 *attB::*pFM17 (P*_nfsA_*::*mCherry*), Km^R^, Tc^R^ | This study |
| PH1244 | Mxan_3227::pFM44, Km^R^ | This study |
| PH1225 | PH1244 *attB*::pFM17 (P*_nfsA_*::*mCherry*) , Km^R^, Tc^R^ | This study |
| DK5208 | DK1622 *csgA*::Tn*5*-132 ΩLS205, Tc^R^ | [4] |
| PH1226 | DK5208 *attB::*pAL4 (P*_nfsA_*::*mCherry*), Tc^R^, Km^R^ | This study |
| ***E. coli* strains** |  |  |
| TOP10 | Host for cloning [F^–^ *mcrA* Δ(*mrr-hsdRMS-mcrBC*) Φ80*lacZ* Δ*M15* Δ*lacX74 deoR recA1 arsD139* Δ(*ara-leu*)*7697 galU galK rpsL* (Str^r^) *endA1 nupG*] | Invitrogen |
| **Plasmids** |  |  |
| pCR2.1-TOPO | cloing vector, Km^R^ | Invitrogen |
| pFM44 | 311-bp internal fragment of Mxan_3327 in pCR2.1-TOPO | This study |
| pBJ114 | Backbone for in-frame deletions; *galK*, Km^R^ | [5] |
| pFM20 | pBJ114 ∆(*nfsA*-*H*) | This study |
| pSWU30 | backbone for Mx8 phage *attP*, TcR | [6] |
| pSL8 | derivative of pSWU30; P*_pilA_*::*gfp*, Km^R^ | S. Leonardy and L. Søgaard-Andersen, unpublished |
| pAL4 | pSL8 derivative P*_nfsA_*::*mCherry*, Km^R^ | This study |
| pAL8 | pAL4 derivative P*_nfsC_*::*mCherry*, Km^R^ | This study |
| pFM17 | pAL4 derivative P*_nfsA_*::*mCherry*, Tc^R^ | This study |
| pFM16 | pAL4 derivative P*_pilA_*::*mCherry*, Km^R^ | This study |
| pFM18 | pSL8 derivative empty control vector, Km^R^ | This study |
|  |  |  |

References

1. Kaiser D: **Social gliding is correlated with the presence of pili in *Myxococcus xanthus***. *Proc Natl Acad Sci U S A* 1979, **76**(11):5952-5956.

2. Thony-Meyer L, Kaiser D: ***devRS*, an autoregulated and essential genetic locus for fruiting body development in *Myxococcus xanthus***. *J Bacteriol* 1993, **175**(22):7450-7462.

3. Sogaard-Andersen L, Slack FJ, Kimsey H, Kaiser D: **Intercellular C-signaling in *Myxococcus xanthus* involves a branched signal transduction pathway**. *Genes Dev* 1996, **10**(6):740-754.

4. Kroos L, Kaiser D: **Expression of many developmentally regulated genes in *Myxococcus* depends on a sequence of cell interactions**. *Genes Dev* 1987, **1**(8):840-854.

5. Julien B, Kaiser AD, Garza A: **Spatial control of cell differentiation in *Myxococcus xanthus***. *Proc Natl Acad Sci U S A* 2000, **97**(16):9098-9103.

6. Wu SS, Kaiser D: **Markerless deletions of *pil* genes in *Myxococcus xanthus* generated by counterselection with the *Bacillus subtilis sacB* gene**. *J Bacteriol* 1996, **178**(19):5817-5821.
