# Supplementary material for: Global transcriptome analysis of spore formation in Myxococcus xanthus reveals a locus necessary for cell differentiation
Source: BMC Genomics. 2010 Apr 26;11:264. doi: 10.1186/1471-2164-11-264 (PMC2875238; doi:10.1186/1471-2164-11-264)
Supplement: Additional file 4 — Primers used for real-time PCR analysis. The list of primers and associated sequences used for real-time PCR analysis (Additional File 1) and reverse transcriptase PCR analysis (Figure 5) in this study. [file 1471-2164-11-264-S4.DOC]

Primer sequences used for PCR amplification of cDNA in this study.

Real-time PCR

*sigB*

forward ATCCGCCTCATCTCCTACGC

reverse CGGGCCAGACTGAAGAACAG

product size: 128 bp

*sigC*

forward TCAACCAGTACCCGCTGCTC

reverse CTTCATCAGGCCGATGTTCG

product size: 194 bp

*mspC*

forward AGAAGCTCATCGCCGCAGTC

reverse GATGCCTGTCACGTCCTTGG

product size: 167 bp

*prU*

forward ACCTGAACGTCACCGCCAAC

reverse TGCCGAACAGGTCGATGC

product size: 129 bp

Mxan_5543

forward AGGGGCTCACGCGCTACTAC

reverse CCTGCACGTCCAGTTCGAG

product size: 153 bp

*atpE*

forward GGGTGCCGGTCTCTCCATC

reverse AAACGACCAGCGCGAACAG

product size: 167 bp

*devR*

forward GCCTCACCGAGGGAAACATC

reverse TGGCTCCTGCTCATTCAAGC

product size: 122 bp

Reverse transcriptase PCR

*nfsB-C*

forward CGAACAAGAAGATGCCCGGTCACTACG

reverse GTACACGCGACTGTCCTGGGGGAC

*nfsC-D*

forward AGTCGCTGTATCGCCCGAAG

reverse GCAATCCAGCACCCACTGAC

*nfsE-F*

forward CTGGGCAGGGCACTGATG

reverse CATGCTCAACCTCCCAATGC

*nfsG-H*

forward GGAGGGTGAGCGACGGTAGCG

reverse CGGTCCATGACGCGCACGTGG
